# Time to Marketing of Generic Drugs After Patent Expiration in Canada

**DOI:** 10.1001/jamanetworkopen.2021.1143

**Published:** 2021-03-11

**Authors:** Joel Lexchin

**Affiliations:** 1 School of Health Policy and Management, York University, Toronto, Ontario, Canada

## Abstract

This cross-sectional study uses marketing data to assess the time from patent expiration of brand name drugs to marketing of generic drug forms.

## Introduction

Pharmaceutical companies argue that 20-year patent protection is not long enough to compensate for the time spent in drug development and the regulatory process. Patents in the United States can be extended for up to 5 years or a maximum of 14 years after US Food and Drug Administration approval.^1^ Canada offers up to 2 extra years of patent protection.^2^ One previous Canadian study examined the time to generic competition but used approval dates rather than actual marketing dates.^3^ The objectives of this study using marketing dates were to:

1. Calculate the percentage of drugs sold in Canada that have generic or biosimilar competition (hereinafter referred to as *generic competition*).
2. Calculate the following times: brand name marketing to patent expiration, patent expiration until generic marketing, and brand name to generic marketing for drugs with generic competition.
3. Calculate the total monopoly for drugs without generic competition and examine if there are factors associated with this time.

## Methods

For this cross-sectional study, a search of the Health Canada database^4^ on December 9, 2020, generated a list of drugs for which patents have expired, in addition to expiration dates of patents and data exclusivity. Patents expired between July 1, 2014, and October 31, 2020. Health Canada annual reports and the Drug Product Database were used to determine the type of drug review (standard or expedited), type of drug (biological product or small-molecule drug), and marketing dates for brand name and generic drugs. The earliest generic marketing date was used. Because all data were publicly available, the study was not required to be submitted to the Office of Research Ethics at York University for ethics approval and informed patient consent. This study followed the Strengthening the Reporting of Observational Studies in Epidemiology (STROBE) reporting guideline for cross-sectional studies.

The number and percentage of drugs with and without generic competition and the various periods were calculated. A drug’s time on the market without competition was compared on the basis of type of drug and type of drug review. Two-sided *t* tests with *P* < .05, the χ^2^ test, and the Fisher exact test were used to test for statistical significance (*P* < .05 for both χ^2^ and Fisher exact tests). Prism version 9.0 (GraphPad) was used for the analysis.

## Results

A total of 121 drugs were available for analysis (Figure). Twenty-nine drugs (24.0%) (all small-molecule drugs) had generic competition, and 92 drugs (76.0%), including all 25 biological products, did not (Table). For drugs with generic competition, the mean time for market exclusivity until patent expiration was 8.08 years (95% CI, 7.94-8.22 years). The mean time from patent expiration until generic drug marketing was 1.53 years (95% CI, 0.87-2.19 years), for a mean difference of 9.61 years (95% CI, 8.92-10.29 years) between the start of brand name and generic drug marketing.

**Figure.**
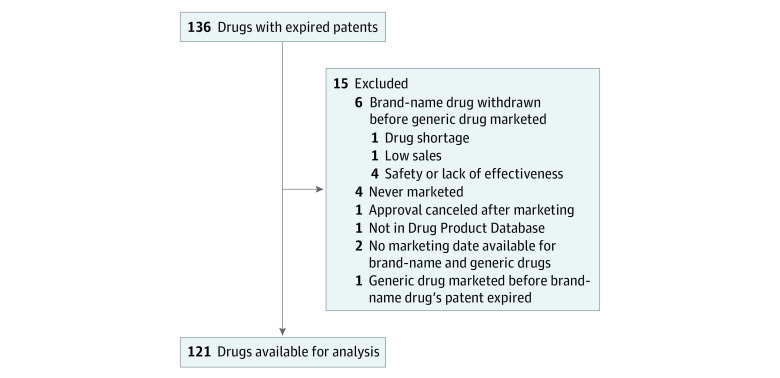
Selection of Drugs for Analysis

**Table.** Type of Drug and Review and Presence or Absence of Generic Competition for 121 Drugs With Expired Patents

	Drug type, No.^a^		Review type, No.^b^	
	Biological product	Small molecule	Standard	Expedited
Generic competition	0	29	24	5
No generic competition	25	67	61	31

^a^ *P* < .001 (by Fisher exact test) for type of drug.

^b^ *P* = .09 (by χ^2^ test) for type of review.

Drugs without generic competition were marketed for a mean of 10.99 years (95% CI, 10.58-11.39 years). Biological products were marketed for a mean of 11.52 years (95% CI, 10.79-12.24 years) compared with a mean of 10.79 years (95% CI, 10.30-11.28 years) for marketing of small-molecule drugs (*P* = .11, *t* test). Drugs with an expedited review were on the market for a mean of 11.91 years (95% CI, 11.18-12.64 years) compared with a mean of 10.52 years (95% CI, 10.06-10.97 years) for drugs with a standard review (*P* = .001, *t* test). Data protection expired before patent protection for all 121 drugs. No drugs had additional patent protection.

## Discussion

Almost one-fourth of the study drugs had generic competition, with an additional 1.53 years from patent expiration until a generic drug was marketed. The presence of biological products may account for the 11-year monopoly marketing time of drugs without generic competition. Canadian market exclusivity periods are shorter than the 12.5 years in the United States,^5^ possibly owing to faster US Food and Drug Administration approval times and the delay in filing for regulatory approval in Canada.^6^ This study has some limitations. Much of the data were from a secondary analysis of Canadian government databases, but no formal evaluation of their quality or validity has been conducted. The percentage of drugs that eventually have generic competition and the length of monopoly sales time should be taken into consideration in future requests for patent term extension.

## References

[1] Beall RF, Darrow JJ, Kesselheim AS. Patent term restoration for top-selling drugs in the United States. Drug Discov Today. 2019;24(1):20-25. doi:10.1016/j.drudis.2018.07.006 30055271

[2] Health Canada. Guidance document: certificates of supplementary protection. December 29, 2020. Accessed November 24, 2019. https://www.canada.ca/en/health-canada/services/drugs-health-products/drug-products/applications-submissions/guidance-documents/register-certificates/certificate-supplementary-protection-regulations.html

[3] Lexchin J. Market exclusivity time for top selling originator drugs in Canada: a cohort study. Value Health. 2017;20(8):1139-1142. doi:10.1016/j.jval.2017.05.004 28964446

[4] Government of Canada. Register of innovative drugs. Accessed December 9, 2020. https://www.canada.ca/en/health-canada/services/drugs-health-products/drug-products/applications-submissions/register-innovative-drugs.html

[5] Beall RF, Darrow JJ, Kesselheim AS. A method for approximating future entry of generic drugs. Value Health. 2018;21(12):1382-1389. doi:10.1016/j.jval.2018.04.1827 30502781

[6] Centre for Innovation in Regulatory Science. R&D Briefing 77: New Drug Approvals in Six Major Authorities 2010-2019: Focus on Facilitated Regulatory Pathways and Internationalisation. Centre for Innovation in Regulatory Science; 2020.

